# Carbon footprint and environmental impact of poultry manure valorization as organic composts

**DOI:** 10.1016/j.psj.2026.106457

**Published:** 2026-01-18

**Authors:** Yu-Ning Chen, Yen-Ching Lin, Shu-Yuan Pan, Ching-Hsun Chang, Tsai-Chi Kuo

**Affiliations:** a Department of Bioenvironmental Systems Engineering, College of Bioresources and Agriculture, National Taiwan University, Taipei City, 10617 Taiwan, ROC; b Department of Industrial Management, National Taiwan University of Science and Technology, Taipei City, 10617 Taiwan, ROC; c Agricultural Net-Zero Carbon Technology and Management Innovation Research Center, College of Bioresources and Agriculture, National Taiwan University, Taipei City, 10617 Taiwan, ROC; d Department of Technology Application and Human Resource Development, National Taiwan Normal University, Taipei City, 10617 Taiwan, ROC

**Keywords:** Poultry manure, Life cycle assessment, Circular agriculture, Environmental impact, Composting, SDG 12

## Abstract

Valorization of poultry manure (PM) has become increasingly important due to the rapid expansion of poultry production, with composting being the most widely used approach to convert PM into organic fertilizer (OF). Conventional composting (CC) typically requires approximately 40 days for manure maturation, whereas high-temperature microorganism decomposition (HMD) can reduce the process to about 7 days. This study quantifies the life-cycle carbon footprint and environmental impacts of OF produced via CC and HMD (i.e., CCOF and HMDOF) using field-measured data. The results show that carbon footprint of CCOF is ∼684 kg-CO_2_e per metric tonne (Mt), while that of HMDOF is ∼801 kg-CO_2_e per Mt, approximately 17% higher. The dominant emission source in both systems is direct methane emission, which primarily produced under anaerobic conditions; therefore, enough aeration and frequent turning during composting are essential strategies to reduce GHG emissions. The higher carbon footprint of HMDOF is primarily attributed to the substantial electricity consumption, highlighting the need for energy optimization or a transition to green energy systems. Beyond GHG emissions, this study also examines several environmental impacts, including acidification, freshwater ecotoxicity, fossil resource use, terrestrial eutrophication, and water use. The freshwater ecotoxicity impact in HMDOF reaches ∼886 CTUe/Mt, compared to ∼269 CTUe/Mt in CCOF, approximately four times higher. Acidification impact is also eight times greater. Resource use in HMDOF is also significantly elevated, at ∼3,310 MJ/Mt, compared to only ∼249 MJ/Mt for CCOF. In addition, this study incorporates a sensitivity analysis on two critical parameters: electricity consumption and composting emission. In CCOF, the sensitivity analysis shows that composting emissions are the primary driver of impact variability, leading to 12–19% changes in carbon footprint, acidification, and terrestrial eutrophication when composting emissions shift by ±20%. Conversely, HMDOF responds more strongly to changes in electricity consumption, with the evaluated impact categories showing 8–17% variation under a ±20% change in electricity use. These findings underscore the importance of evaluating environmental performance from multiple perspectives when pursuing faster and more advanced composting technologies. A comprehensive assessment ensures that innovations align with the principles of circular economy and environmental sustainability.

## Nomenclature

AD: Anaerobic Digestion
BOD: Biochemical Oxygen Demand
CC: Conventional Composting
CCOF: Conventional Composting Organic Fertilizer
COD: Chemical Oxygen Demand
CTUe: Comparative Toxic Unit for Ecosystems
C/N: Carbon-to-Nitrogen
NMVOC: Non-Methane Volatile Organic Compounds
GHG: Greenhouse Gas
HMD: High-Temperature Microbiological Decomposition
HMDOF: High-Temperature Microorganism Decomposition Organic Fertilizer
HTC: Hydrothermal Liquefaction
HTL: Hydrothermal Carbonization
K: Potassium
LCA: Life Cycle Assessment
MJ: Megajoules
MT: Metric Tonne
MMT: Million Metric Tons
MOE: Ministry of Environment
N: Nitrogen
P: Phosphorous
PM: Poultry Manure
SS: Suspended Solids

## Introduction

Globally, agricultural production systems generate vast amounts of solid waste (Kamusoko et al., 2021). According to the statistic of FAOSTAT (2024), global chicken manure production amounts to 1,112 ± 75 million metric tons (MMT) per year. In particular, the rise in meat production to meet growing human consumption has led to an increase in environmental pollution, especially from the management of livestock manure (Saleem et al., 2023). In the United States, greenhouse gas (GHG) emissions from livestock manure management were estimated at ∼83.4 MMT CO_2_-eq in 2021, with poultry manure (PM) accounting for ∼5.2 MMT CO₂-eq (Del Grosso et al., 2008). Improper PM management, such as indiscriminate dumping or open burning, leads to GHG emissions to air, emissions of nitrogen (N) and phosphorous (P) to surface water (Bolan et al., 2010), economic losses (Szogi and Vanotti, 2009), human health-related problems (Gay et al., 2003), and broader threats to food and energy security. As a result, transforming PM into a renewable and valuable resource has become a critical issue for sustainable agriculture. Consisting of digested and transformed biomass that is rich in N, P, and potassium (K), adopting PM-based fertilizer is a big step toward the agricultural closing-loop (Huang et al., 2011; Jia et al., 2025; Mitrovic et al., 2022).

PM is commonly treated with waste eggs, chicken carcasses, and spent mushroom by conventional composting (CC) processes to produce organic compost fertilizers. However, the high moisture content of PM (approximately 75∼90%) and its low porosity hinder adequate aerobic microbial activity (Shan et al., 2021; Wang et al., 2021). The low carbon-to-nitrogen (C/N) ratio of PM also leads to nitrogen loss via ammonia emissions and inhibits the growth and reproduction of beneficial microorganisms (Li et al., 2022; Zhou et al., 2017). Recent studies have investigated the integration of effective microorganisms and biochar into the CC process to enhance microbial activity, improve porosity, increase composting temperature, adjust C/N ratio and pH, and ultimately improve both yield and quality of final products (Iriti et al., 2019; Sanchez-Monedero et al., 2018; Wang et al., 2017). Although CC is widely applied, it requires a relatively long maturation period (e.g., ∼40 days). In contrast, high-temperature microbiological decomposition (HMD) processes are inoculated with specific aerobic microorganisms that rapidly increase the temperature, thereby creating a more favorable environment for microbial activity. These elevated temperatures are effective in eliminating various pathogens, insect eggs, and weed seeds. Through microbial self-metabolism, part of the organic matter is mineralized into simpler inorganic substances with associated energy release, while the remaining fraction is converted into new microbial biomass. Therefore, the HMD process significantly reduces the maturation period to 5∼7 days, offering strong incentives for stakeholders to adopt this technology.

Selecting appropriate technologies for PM valorization requires not only evaluating treatment efficiency and product quality, but also assessing environmental performance. Several studies have investigated the valorization of PM as bioenergy through various technologies. For instance, Scarlat et al. (2018) estimated that livestock and PM in Europe could produce up to 26 billion m^3^ biomethane in Europe, while also analyzing its spatial distribution across the continent. Akhtar Usmani and Khan (2025) also studied the biomass potential for energy utilization, with an emphasis on the transportation sector. In addition to renewable energy applications, other studies have focused on converting PM into biochar for use as a soil amendment or carbon sink (Al-Zoubi et al., 2024; Lee et al., 2024). To the best of our knowledge, although composting PM into organic fertilizer remains the most widespread valorization strategy, the relevant research of comparing different composting methods across their entire life cycle remains limited. For example, Kiss et al. (2022) assessed global warming potential, acidification potential and eutrophication potential of composting and utilizing broiler PM-based organic fertilizer through a life cycle assessment (LCA) framework. Despite these progresses, few studies have focused on the environmental impacts and carbon footprints of PM valorization and product from a life-cycle perspective, which is a notable research gap.

To address this research gap, this study evaluates two PM composting fertilizers, i.e., conventional composting organic fertilizer (CCOF) and high-temperature microbiological decomposition organic fertilizer (HMDOF), within a cradle-to-gate system boundary. Using field-measured data, the carbon footprint and environmental impacts of CCOF and HMDOF are quantified. GHG emission and environmental impact hotspots across life-cycle stages are identified, with sensitivity analysis conducted to address parameter uncertainty. The results provide a comprehensive understanding of the life-cycle performance of advanced poultry manure management strategies and highlight their potential role in mitigating environmental impacts of agriculture through integrated livestock–crop systems.

## Methods

By applying LCA, potential environmental hotspots throughout the process can be identified, serving as key targets for improvement. LCA in this study was conducted in three steps: (1) selecting the target product, (2) collecting production data, and (3) quantifying both the carbon footprint and the environmental impact. Two essential elements in any LCA study are the system boundary and the declared unit. A well-defined and consistent system boundary and declared unit ensure the reliability and comparability of results between different products or systems. The system boundary of the assessment in this study spans from cradle to gate. The methodological framework follows the guidelines of the International Organization for Standardization, specifically ISO 14067:2018 and ISO 14064:2018 (ISO, 2018a; ISO, 2018b).

### Scope and scenario set-up

In this study, two composting methods for PM valorization, i.e., CC and HMD, were evaluated. In CC processes, the compost maturation period is approximately 40 days. During this period, a mechanical turner is used to regularly aerate the composting materials, ensuring enough oxygen supply for aerobic microorganism activity. After 40 days, the mature compost is packaged and then transported to end users as organic fertilizer. Throughout the composting process, gaseous emissions are generated, mainly including CO_2_, CH_4_, and N_2_O. In addition to gaseous emissions, wastewater is produced from the scrubber tower used to treat odors generated during processing. Wastewater quality parameters, including biochemical oxygen demand (BOD), chemical oxygen demand (COD), and suspended solids (SS), were determined in accordance with the National Institute of Environmental Analysis standard methods: BOD (NIEA W510.56B), COD (NIEA W517.53B), and SS (NIEA W210.58A). The average measured concentrations of BOD, COD, and SS in the wastewater were approximately 7.5, 77.9, and 20.5 mg/L, respectively.

The HMD process enhances composting efficiency by introducing specific aerobic microbial strains. In HMD, microbial activity rapidly raises the composting temperature to 60 °C, which further increases to 70∼80 °C and remains at that level for several days. Unlike CC, wastewater generated in HMD is collected and recycled as high-nitrogen fluid fertilizer. Notably, the compost maturation period in HMD is ∼7 days, significantly shorter than the 40-day period required for conventional composting, making the process approximately five times faster.

**Fig. 1:**
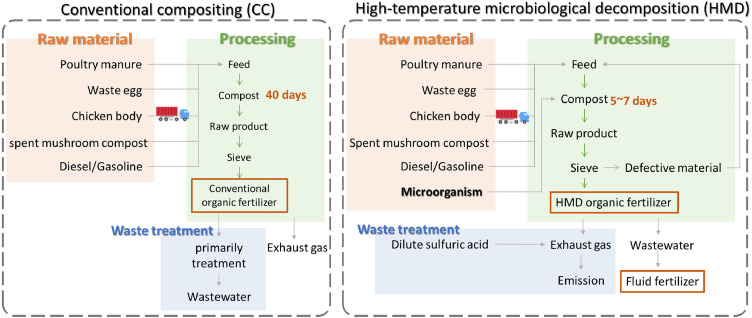
System boundary of conventional composting (CC) and high-temperature microorganism decomposition (HMD) processes for poultry manure valorization.

As illustrated in Fig. 1, the scope of this study includes four main stages: (1) raw material processing, (2) transportation, (3) organic fertilizer processing, and (4) waste treatment. The functional unit of LCA is declared to be “producing one metric tonne (Mt) of organic fertilizer such as CCOF and HMDOF”. Due to data collection limitations, the timeframes for the two composting methods differ. For CC, data were collected from January 2022 to December 2022; for HMD, data were gathered from October 2023 to March 2024. During the period considered, the CC process treated 9,305 Mt of raw PM while producing 3,655 Mt of organic fertilizer. In comparison, HMD processed 3,503 Mt of raw PM while producing 1,245 Mt of organic fertilizer and 180 m^3^ high-nitrogen liquid fertilizer. The allocation in HMD is based on weight balance.

### Life cycle inventory (LCI)

In this study, the LCI data were collected from two local manufacturers located in Pingtung and Tainan, Taiwan as the representative cases of CC and HMD processes, respectively. These two chicken farms mainly raise laying hens. Table 1 presents the detailed LCI of CC and HMD processes for producing the organic fertilizer. The activity data for electricity use, fuel consumption, auxiliary materials, and wastewater treatment were obtained from the manufacturer’s production records and on-site operational information. This classification includes raw materials, energy and resource use, and transportation. In the raw material phase, all materials used by the manufacturer to produce organic fertilizer are accounted for. However, certain items are excluded if they are considered long-term capital goods. In the energy and resource use phase, all resources consumed during production are included. Electricity is primarily used to operate equipment such as processing machines, exhaust fans, and ventilation systems. Diesel and gasoline are used to power the compost turner, internal transport vehicles, and other factory machinery.

**Table 1:** Life cycle inventory of conventional composting (CC) and high-temperature microorganism decomposition (HMD) processes.

Item	Energy and Material	Unit	Activity data	
			CC	HMD
Raw material input	Poultry manure & biowaste^1^	Tonne	9,305	3,131
	Spent mushroom compost	Tonne	824	325
	Plastic bag	Tonne	0.6	-
	Microorganism	Tonne	-	3.4
	Stretch wrap	Tonne	-	0.5
	Dilute sulfuric acid	Tonne	-	50.7
Energy and resource use	Electricity	kWh	28,189	409,536
	Diesel	L	35,436	2,000
	Gasoline	L	-	300
	Tap water	Mt	9	150
Transport	Spent mushroom compost	tkm	24,720	325
	Plastic bag	tkm	40.8	-
	Microorganism	tkm	-	3.4
	Stretch wrap	tkm	-	0.5232
Output	Organic fertilizer (OF) product	Tonne	3,655	1,245
	Wastewater	m^3^	9	-
	Liquid fertilizer	L	-	180,000

1 Poultry manure & biowaste are generated from chicken farm.

### Carbon footprint

According to ISO 14064 (ISO, 2018a) and 14067 (ISO, 2018b), GHG emission inventories are categorized into six categories: (1) direct GHG emissions and removals, (2) indirect GHG emissions from imported energy, (3) indirect GHG emissions from transportation, (4) indirect emissions from upstream manufacturing including electricity and resource processing, (5) indirect emissions from product use, and (6) indirect emissions from all other sources. Based on the system boundary shown in Fig. 1, the carbon footprint is aggregated into four categories. Category 1 comprises direct emissions from composting (N_2_O and CH_4_), wastewater discharge, and other fuel utilization during the process. Category 2 covers emissions from on-site electricity use. Category 3 includes emissions from the transportation of raw materials to the composting facilities. Category 4 represents upstream manufacturing processes, including electricity generation and diesel fuel production. The product carbon footprint is calculated by Eq. (1):

(1) Carbonfootprint=Activitydata×Emissionfactor×Globalwarmingpotential(GWP)

Table 2 summarizes the GHG emission factors used in this study. The emission factors of composting processes were obtained from the literature cited in the National Greenhouse Gas Inventory, i.e., 7.85 kg-CH_4_, and 0.07 kg-N_2_O per metric ton of raw manure (MOA Taiwan, 2024; Wang and Ma, 2002). CO_2_ emissions from composting processes are considered part of natural biogenic carbon cycle and therefore excluded from the carbon footprint calculation (IPCC, 2006). The remaining GHG emission factors of energy and material were primarily referenced from the Carbon Footprint Information Platform established by the Ministry of Environment (MOE), Taiwan, ensuring that the data reflect local conditions. Since no localized reference exists for emissions associated with microorganism, the emission factor for microorganism were obtained from the Ecoinvent version 3.0 database (Ecoinvent, 2023). The GWP values are adopted from the IPCC Sixth Assessment Report (IPCC, 2023), with values of 273 kg CO_2_-eq for N_2_O and 29.8 kg CO_2_-eq for CH_4_ over a 100-year time horizon.

**Table 2:** Emission factors of greenhouse gases for each activity used in this study.

Scope	Category	Item	EF^1^	Unit	Description	Reference
1	1	Composting processes	0.070	kg-N_2_O/ tonne	GHG emissions were sampled during composting treatment of laying-hen manure using a gas collection hood. The collected gas samples were analyzed by GC to quantify CH_4_, N_2_O, and CO_2_.	Wang and Ma (2002)
			7.850	kg-CH_4_/ tonne		Wang and Ma (2002)
		Wastewater treatment processes	0.600	kg-CH_4_/ kg-BOD	The maximum amount of CH_4_ that can be produced from a given quantity of organics (as expressed in BOD or COD) in the wastewater	Taiwan MOE (2022)
			0.250	kg-CH_4_/ kg-COD		Taiwan MOE (2022)
		Diesel use	2.617	kg-CO_2_e/ L	Only on-site stationary source use is considered.	Taiwan MOE (2022)
		Gasoline use	2.316	kg-CO_2_e/ L	Only on-site mobile source use is considered.	Taiwan MOE (2022)
2	2	Electricity	0.495	kg-CO_2_e/ kWh	Covers raw material extraction, feedstock transportation, fuel refining and combustion, grid transmission.	Taiwan MOE (2022)
3	3	Heavy truck (diesel)	0.131	kg-CO_2_e/ tkm	GHG emissions from diesel fuel used by domestic heavy trucks for freight transport, including emissions associated with fuel production, distribution, and fuel consumption during vehicle operation.	Taiwan MOE (2022)
		Light truck (diesel)	0.587	kg-CO_2_e/ tkm	GHG emissions from diesel fuel used by domestic light trucks for freight transport, including emissions associated with fuel production, distribution, and fuel consumption during vehicle operation.	Taiwan MOE (2022)
	4	Microorganism	1.990	kg-CO_2_e/ kg	Fermentation including materials, energy uses, infrastructure, and emissions.	Wernet et al. (2016)
		Stretch wrap	2.020	kg-CO_2_e/ kg	Mixed and melted plastic feedstock, extract to cool, then wound into a cylindrical roll and packaged. Covered from cradle-to-gate.	Taiwan MOE (2022)
		Tap water	0.233	kg-CO_2_e/ m^3^	From pumping water at source into the conveyance channel, the process includes water conveyance and treatment, and ends with distribution to end users.	Taiwan MOE (2022)
		Dilute sulfuric acid	0.289	kg-CO_2_e/ kg	Sulfur is burned to produce SO_2_, which is then catalytically converted into sulfuric acid. Covered from cradle-to-gate.	Taiwan MOE (2022)
		Electricity	0.111	kg-CO_2_e/ kWh	Covers distribution to the end-user outlet, and waste treatment.	Taiwan MOE (2022)
		Diesel	0.673	kg-CO_2_e/ L	From crude oil extraction through delivery to domestic market. The heat value conversion factor is 35.17 MJ/L	Taiwan MOE (2022)
		Gasoline	0.604	kg-CO_2_e/ L	From crude oil extraction through delivery to domestic market. The heat value conversion factor is 32.66 MJ/L	Taiwan MOE (2022)

1 EF = Emission factor.

### Environmental impact

This study adopts the Environmental Footprint version 3.1 methodology developed by the European Commission (Andreasi Bassi et al., 2023), using SimaPro 9.5 (SimaPro, 2023) and the ecoinvent V3.0 database (Ecoinvent, 2023). While this methodology encompasses 16 impact categories, this study focuses on six key categories relevant to PM composting including acidification, freshwater ecotoxicity, terrestrial eutrophication, photochemical ozone formation, fossil resource use, and water use. Acidification is measured in mol H^+^ eq and is associated with the decline of forests and increased fish mortality. The main contributions come from combustion in electricity, heat production, and transport. Freshwater ecotoxicity is measured in comparative toxic unit for ecosystems (CTUe) and refers to potential toxic impacts on ecosystem and aquatic living organisms. Photochemical ozone formation evaluates the potential contribution to ground-level ozone formation and is converted into the equivalent of kilograms of non-methane volatile organic compounds (kg NMVOC eq). Fossils resource use quantifies the consumption of fossil resources and is expressed in megajoules (MJ). Water use is expressed in cubic meters of water and is related to the local water scarcity. Finally, terrestrial eutrophication is caused by substances containing nitrogen and phosphorus, which promote growth of algae or specific plants and is expressed in equivalent of moles of nitrogen (mol N eq) (Andreasi Bassi et al., 2023).

### Sensitivity analysis methods

To address the uncertainty of each parameter in LCA calculations, sensitivity analysis is an essential part to enhance the robustness of the model (Bala et al., 2010; ISO, 2018a; Wei et al., 2015). In this study, based on previous investigate on environmental impacts, electricity consumption and composting emissions are identified as emission hotspots. To better reflect real-word variability, continuous sensitivity variation is applied over a range of 5–20%, rather than using fixed variation levels. The resulting sensitivity indices for environmental impacts provide insights into the relationship between those parameters and their corresponding environmental outcomes.

## Results and discussion

### Carbon footprint analysis

**Fig. 2:**
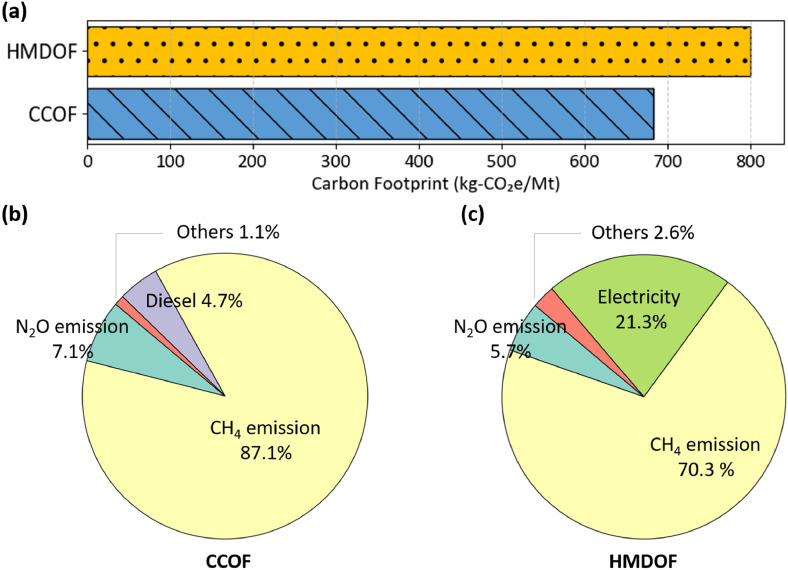
Carbon footprint of CCOF and HMDOF: **(a)** carbon footprint, **(b)** emission sources from CCOF, and **(c)** emission sources from HMDOF. Abbreviations: CCOF = Conventional composting organic fertilizer; HMDOF = high-temperature microbiological decomposition organic fertilizer.

As shown in Fig. 2**(a)**, based on the on-site data calculated from LCA perspective, the carbon footprint of CCOF is ∼684 kg-CO_2_e per Mt, while that of HMDOF is ∼801 kg-CO_2_e per Mt. Although HMDOF can reach maturity in one-fifth the time required for CCOF, its carbon footprint is approximately 17% higher. Fig. 2**(b) and 2(c)** illustrate the emission sources associated with CCOF and HMDOF, respectively. In both cases, direct emissions from composting process are the dominant contributors, particularly CH_4_. CH_4_ emissions account for 87.1% of the total carbon footprint of CCOF and 70.3% of HMDOF. In aerobic composting systems, most methane is expected to be oxidized into CO_2_, which helps mitigate GHG emissions (Walling and Vaneeckhaute, 2020). Beck-Friis et al. (2000) reported that CH_4_ emissions tend to increase as oxygen availability decreases. N_2_O emissions arise from either incomplete ammonium oxidation or (de)nitrification processes. Therefore, it can be inferred that both CH_4_ and N_2_O emissions result from incomplete oxidation composting. In addition, a notable difference is the significant contribution of electricity-related emissions in HMDOF. According to the LCI data, electricity consumption in HMDOF is approximately 40 times higher than that of CCOF. This is primarily due to the use of automated control systems and smart panels in the HMD composting system. Furthermore, to mitigate odor emissions, the HMDOF system operates under negative pressure, which requires continuous electricity input to manage air circulation and exhaust gas control. In contrast, conventional composting relies less on electricity-driven devices, resulting in a smaller contribution of electricity to the total carbon footprint.

**Fig. 3:**
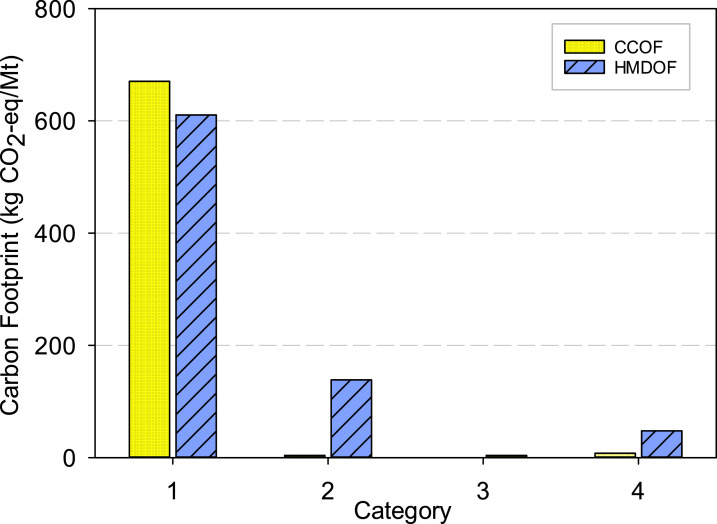
Carbon footprint contributions categorized by emission source for CCOF and HMDOF. Abbreviations: CCOF = Conventional composting organic fertilizer; HMDOF = high-temperature microbiological decomposition organic fertilizer. Category 1: Direct emissions from manufacturing processes; Category 2: Emissions from energy and resource consumption, Category 3: Emissions from transportation; Category 4: Emissions from upstream manufacturing including electricity and resource processing.

Based on the system boundary, the carbon footprint is aggregated into four major categories, as shown in Fig. 3. Category 1 comprises direct emissions from composting (N_2_O and CH_4_) and wastewater discharge. Category 2 covers emissions from on-site electricity use and diesel fuel consumption. Category 3 includes emissions from the transportation of raw materials to the composting facilities. Category 4 represents upstream manufacturing processes, including electricity generation and diesel fuel production. For both CCOF and HMDOF, Category 1 contributes the most to emissions, reaching approximately 646 kg CO₂-eq and 610 kg CO₂-eq per tonne, respectively, reflecting that incomplete aerobic stabilization during PM composting makes CH₄ a major direct emission source. In contrast, HMDOF exhibits significantly higher emissions than CCOF in Categories 2 and 4, with Category 2 (energy and resource use) 5.7 times higher and Category 4 (upstream manufacturing) 7.5 times higher, mainly due to electricity processing and consumption. These results confirm that the large carbon footprint difference between CCOF and HMDOF is primarily driven by electricity use, indicating that reducing electricity consumption or switching to green energy is essential for lowering HMDOF emissions.

### Environmental impact analysis

To provide a more comprehensive assessment of environmental sustainability of each product, this study selects representative environmental impact categories to compare the environmental impacts of CCOF and HMDOF. The selected categories include: acidification, freshwater ecotoxicity, photochemical ozone formation, fossils resource use, water use and eutrophication were analyzed. Table 3 presents the stage-wise environmental impact of CCOF and HMDOF. It is evident that consistently exhibits higher environmental impacts across all assessed categories compared to CCOF. Notably, electricity consumption contributes significantly to the overall impact, particularly in the categories of freshwater ecotoxicity and fossil resource use. For HMDOF, electricity accounts for 54% of the freshwater ecotoxicity impact, followed by raw material inputs, such as microorganisms, sulfuric acid and packaging materials, contributing 27%. In terms of fossil resource use, electricity alone is responsible for as much as 87% of the total impact.

**Table 3:** Stage-wise environmental impact assessment of CCOF and HMDOF products (functional unit: per metric tonne of organic fertilizer production).

Impact category	Unit 1	Product	Life-cycle Stage					Total
			Direct Emission	Diesel/ Petrol	Transport	Material Input	Electricity Use	
Ecotoxicity, freshwater	CTUe	CCOF	6.4	216.5	32.6	0.2	13.8	269.4
		HMDOF	6.0	34.4	139.5	223.3	482.6	885.7
Photochemical ozone formation	kg NMVOC-eq	CCOF	0.4	0.1	0.0	0.0	0.0	0.5
		HMDOF	0.4	0.0	0.1	0.1	0.5	1.0
Resource use, fossils	MJ	CCOF	0.0	428.0	55.0	0.3	82.2	565.5
		HMDOF	0.0	67.9	235.6	121.5	2884.6	3309.6
Water use	m^3^ deprived	CCOF	0.0	0.6	0.2	0.0	0.8	1.6
		HMDOF	0.0	0.1	1.1	41.0	27.2	69.3
Eutrophication, terrestrial	mol N-eq	CCOF	0.8	0.1	0.1	0.0	0.0	1.0
		HMDOF	0.7	0.0	0.4	0.3	1.5	2.9
Acidification	mol H^+^-eq	CCOF	0.1	0.1	0.0	0.0	0.0	0.2
		HMDOF	0.1	0.0	0.1	0.6	0.8	1.6

1 CTUe = Comparative toxic unit for ecosystems; NMVOC = Non-methane volatile organic compounds; MJ = Megajoule.

**Fig. 4:**
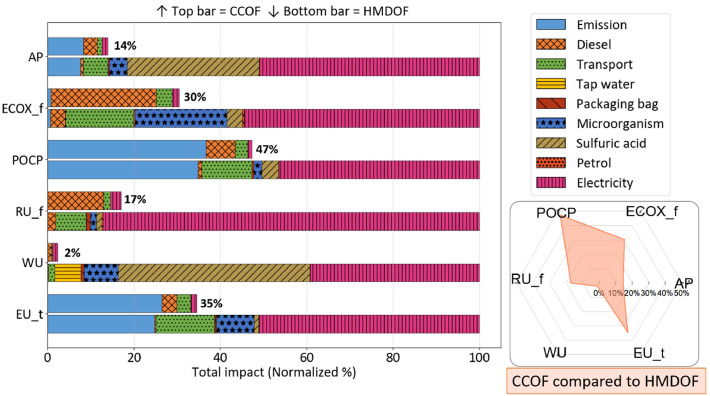
Normalized environmental impact comparison between CCOF and HMDOF. AP: acidification; ECOX_f: ecotoxicity, freshwater; POCP: photochemical ozone formation; RU_f: resource use, fossil; WU: water use; EU_m: eutrophication, marine.

Fig. 4 illustrates the normalized contributions of each component to the environmental impact for both composting systems. The values displayed at the right end of the CCOF bars represent the relative contribution intensity of CCOF compared to HMDOF under each category. In the carbon footprint analysis, direct emissions from the composting process are the dominant contributor, accounting for 95% of total emissions in CCOF and 76% in HMDOF. However, the environmental impact results reveal that the higher overall impact of HMDOF is primarily attributed to its substantial electricity consumption. This highlights a key distinction: while composting emissions dominate in carbon terms, energy use becomes the main differentiator in broader environmental impacts.

The most significant difference between the two systems appears in water use, where CCOF contributes only 2% compared to HMDOF. In the HMDOF system, sulfuric acid and electricity are the primary contributors to water use, accounting for 44% and 39%, respectively, while microorganism cultivation also contributes approximately 8%. Similarly, the acidification impact of CCOF is only 12% of that in HMDOF. Within HMDOF, sulfuric acid and electricity are again dominant sources, contributing ∼31% and ∼51% of acidification potential, respectively. Notably, although microorganisms account for less than 1% of the total carbon footprint, they contribute significantly to other environmental categories: ∼21% freshwater ecotoxicity, and ∼10% water uses and terrestrial eutrophication. For the remaining environmental categories, CCOF exhibits 15–41% of the impact levels observed in HMDOF. Sulfuric acid and electricity dominate the water-related categories. For other categories, the dominant factor is typically electricity. These findings further reinforce the importance of reducing electricity consumption in HMDOF, as it remains a key driver of its elevated environmental impact across multiple categories.

### Sensitivity analysis

**Fig. 5:**
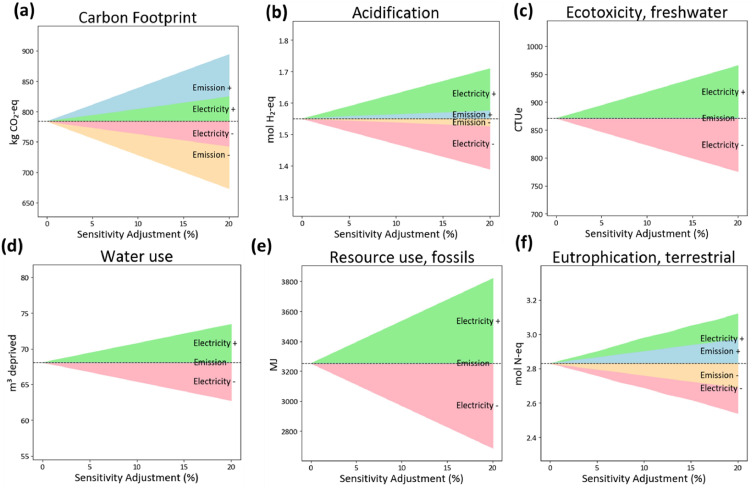
Continuous sensitivity analysis of HMDOF environmental impacts to electricity use and composting-emission variations (±5∼20%).

**Fig. 6:**
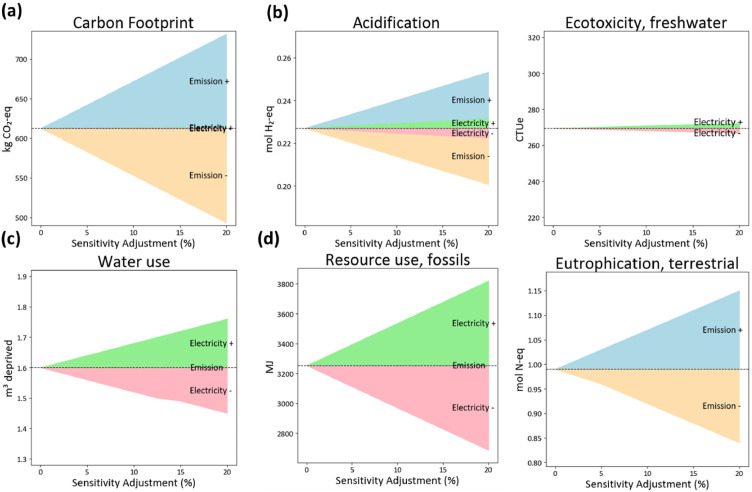
Continuous sensitivity analysis of CCOF environmental impacts to electricity use and composting-emission variations (±5∼20%).

GHG emissions from compost contribute ∼94.2% of the carbon footprint of CCOF and ∼75.8% of HMDOF, highlighting composting as a major hotspot. In addition, electricity use accounts for ∼21.3% of the total emissions of HMOF. Therefore, a sensitivity analysis was conducted by varying both electricity consumption and composting-related emissions. Fig. 5, Fig. 6 show the sensitivity analyses for HMDOF and CCOF, respectively. Note that the carbon footprint results in this section were calculated in SimaPro using emission factors from Ecoinvent v3.1. Therefore, these values may differ slightly from the results reported earlier, which were estimated using domestic data sources. In both figures, the upper and lower bounds represent a ±20% variation in environmental impacts; thus, a larger shaded area indicates a stronger influence of that category on the overall results. Fig. 5 shows that composting emissions have the greatest influence on carbon footprint in HMDOF: a ∼20% change in composting emissions results in a ∼14% variation in carbon footprint. Nonetheless, for other HMDOF impact categories, composting emissions are less influential than electricity. For example, in freshwater ecotoxicity, water use, and fossil resource use, a 20% variation in composting emissions leads to only a 1–2% change. This may be because some emissions are scrubbed prior to release. By contrast, electricity variation shows a stronger effect across several categories. Specifically, a ∼20% change in electricity use leads to a ∼17% variation in fossil resource use. variation. Similarly, for a 20% electricity variation, the sensitivity in terrestrial eutrophication, water use, and acidification reaches 15%, 18%, and 10%, respectively. These results suggest that reducing electricity consumption could yield substantial improvements across multiple environmental impact categories for HMDOF.

Fig. 6 shows the sensitivity analysis for CCOF, since composting dominates the carbon footprint in CCOF, variations in overall impacts are largely driven by changes in composting emissions. Particularly for carbon footprint, acidification, and terrestrial eutrophication, which vary by 19%, 12%, and 15%, respectively, under a ±20% variation. In contrast, electricity-related variations have a relatively minor influence on CCOF. For example, acidification and freshwater ecotoxicity change by only ∼1–2% when electricity use increases by ∼20%. Proper aeration during composting is crucial to minimize CH_4_ formation. CH_4_ is produced through methanogenesis, a biological process occurring under anaerobic conditions. Under aerobic conditions, however, composting primarily generates CO_2_ and H_2_O, both of which have much lower GWP compared to CH_4_. Notably, variations in composting emissions have little to no effect on fossil resource use and freshwater ecotoxicity. Overall, the sensitivity analysis indicates that CCOF is predominantly influenced by composting, particularly through its contribution to carbon footprint, whereas HMDOF is more sensitive to electricity consumption, with fossil resource use being the most affected category.

### Insights into the life cycle perspective of advanced poultry manure management strategies

The LCA provides a comprehensive analysis of both CCOF and HMDOF, covering impact categories such as carbon footprint, acidification, freshwater ecotoxicity, photochemical ozone formation, fossil resource use, water use, and marine eutrophication. The results reveal the merits and drawbacks of the two approaches. HMDOF, with the introduction of microorganisms, can shorten the composting period to 7 days. Additionally, the highly automated facility reduces labor requirements. In contrast, CCOF employs traditional composting, requiring a longer composting period around 40 days; however, due to its low electricity consumption, it shows not only a lower carbon footprint but also reduced impacts across other environmental impact.

**Fig. 7:**
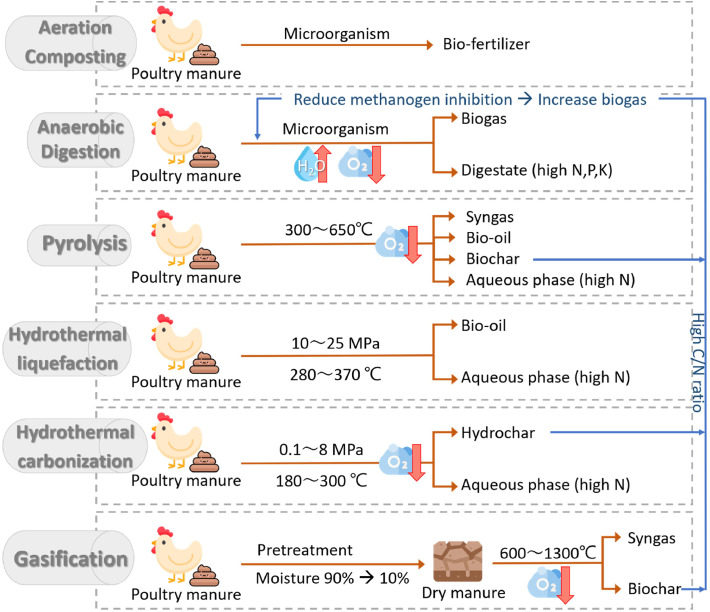
Overview of innovative utilization pathways for chicken manure.

Fig. 7 illustrates the emerging technologies for PM valorization, including aeration composting, anaerobic digestion (AD), pyrolysis, hydrothermal liquefaction (HTL), hydrothermal carbonization (HTC), and gasification. This study extensively discusses how aeration composting transforms PM into bio-fertilizer. Moreover, AD is also a biochemical treatment for PM requiring high-moisture feedstock and low oxygen conditions, which suit PM due to the moisture content of 75∼90%. AD converts PM into biogas, a critical bioenergy, and digestate, a nutrient-rich liquid fertilizer. The other technologies, pyrolysis, HTL, HTC, and gasification, are thermochemical processes that operate at high temperature. Pyrolysis is an oxygen-free process in which biomass thermally decomposes to generate bio-oil and syngas (CO, H_2_, CO_2_, CH_4_), biochar, and nutrient-rich liquid (Iyer et al., 2020). HTL and HTC simulate natural fossil fuel formation under controlled conditions but within a much shorter timeframe. In HTC, the pressure is usually autogenous, increasing with temperature (Funke and Ziegler, 2010). In contrast, HTL requires externally maintained high pressure to keep the material in the liquid phase (Odales-Bernal et al., 2025; Toor et al., 2011). The key difference between these two processes lies in their primary products: HTL mainly produces bio-oil, while HTC yields hydrochar. Both are hydrocarbon-rich products with energy potential. Integrating these approaches by introducing biochar and hydrochar into the AD system is a promising strategy. High concentrations of ammonia, VFA inhibit the activity of methanogens, thereby reducing methane generation (Kwak et al., 2020; Wang et al., 2025). Introducing high C/N ratio materials can reduce ammonia inhibition and enhance methane yield (Fang et al., 2019). Table 4 summarizes the LCA frameworks and assumptions used to analyze PM valorization pathways, highlighting the carbon footprints of each advanced strategies under various scenarios. It shows that aeration composting is often performed as co-composting with lignocellulosic biomass. Furthermore, LCA studies specifically addressing systems with microbial inoculation are still lacking, emphasizing the significance of this study. In addition, thermochemical processes are frequently coupled with power generation, which allows for offsetting fossil-based electricity use—resulting in low or even negative carbon footprints.

**Table 4:** Comparison of carbon footprints of common chicken manure conversion technologies.

Treatment method		LCA Framework and Assumptions				Carbon footprint (kg-CO_2_e)		Reference
		Country	System boundary	Declared unit	BAU	BAU	Conversion technology	
Aeration composting	Windrow composting	Germany	Gate to cradle	1 Mg of raw poultry manure	Direct land use (replacing chemical fertilizer)	-81	216	Kreidenweis et al. (2021)
	Co-composting with mushroom bran	China	Grave to gate	1 Mg of raw poultry manure	Compost with manure mixture (tobacco)	106	75	Zhang et al. (2022)
	Co-composting with mushroom bran and biochar						54	Zhang et al. (2022)
	Co-composting with wheat straw and TAB	China	Gate to Gate	1 L PVC reactor	Co-composting with wheat straw	148	2% TAB: 133 4% TAB: 131 6% TAB: 121 8% TAB: 114 10% TAB: 109	Ren et al. (2019)
AD	CHP	Germany	Gate to cradle	1 Mg of raw manure	storage before distribution	-81	-432	Kreidenweis et al. (2021)
	CHP cogenerated through AD of different types	Turkey	Cradle to cradle	1 MWh of net electricity and heat generated	Natural gas boiler	529	cattle manure +chicken manure: 507; cattle slurry+ chicken manure+ vegetable wastes: 232	Balcioglu et al. (2022)
	Cogeneration with steam-power (37°C)	Singapore	Cradle to Gate	1 Mg of dry raw manure	Direct land application	76	38	He et al. (2022)
	AD with venting biogas.	USA (New York)	cradle to gate	1 Mg of raw poultry manure	Direct Land Application	106	4211	Preuss and You (2024)
	AD with CHP of biogas and land application of digestate.	USA (New York)	cradle to gate	1 Mg of raw poultry manure	Direct Land Application	106	80	Preuss and You (2024)
	Cogeneration with macroalgae (40%) and chicken manure (60%)	Germany	Cradle to Gate	1 MJ of power generation	current production system with energy crops (maize, grass, rye, and chicken manure)	28.9	13.9	Ertem et al. (2017)
Pyrolysis	500°C to produce biochar	Germany	Gate to cradle	1 Mg of raw manure	storage before distribution	-81	-192	Kreidenweis et al. (2021)
	Pyrolysis with biochar, selling bio-oil	USA (New York)	cradle to gate	1 Mg of raw poultry manure	Direct Land Application	106	-874	Kreidenweis et al. (2021)
	Pyrolysis with biochar and selling bio-oil, CHP of syngas	USA (New York)	cradle to gate	1 Mg of raw poultry manure	Direct Land Application	106	−802	Preuss and You (2024)
Gasification	Gasification and produce biochar as byproduct	Singapore	Cradle to Gate	1 Mg of dry raw manure	Direct land application	76	-83	He et al. (2022)

* Abbreviation: CHP = Combined heat and power, AD= anaerobic digestion, TAB= tertiary-amine bentonite.

## Conclusion

This study demonstrates the carbon footprint and environmental impact of two PM composting technologies—conventional composting organic fertilizer and high-temperature microbiological decomposition organic fertilizer. Direct gaseous emissions during composting, particularly CH_4_, were the dominant contributor in both systems, accounting for 95% and 76% of total carbon emissions in CCOF and HMDOF, respectively. CH_4_ is produced by microorganisms under anaerobic conditions, especially at elevated temperatures. During the thermophilic phase, thermophilic methanogens may significantly enhance methane generation (Jäckel et al., 2005). Therefore, continuous turning and aeration are effective strategies for reducing GHG emissions during PM composting. While adopting microorganism in HMD system showed a fivefold reduction in composting time, its carbon footprint was 17% higher than that of CCOF. Further compared in environmental impact, HMDOF consistently exhibited higher impacts, with particularly large gaps in water use (50 times higher) and acidification (8 times higher). These differences were largely attributed to electricity consumption and sulfuric acid use, both of which played significant roles in water-related and acidification impacts. Across other impact categories, CCOF retained 15–41% of the environmental impact levels of HMDOF mostly due to lower electricity consumption, showing its comparative advantage in sustainability. This showing that while technological advancements can enhance processing efficiency, they must also prioritize energy efficiency to avoid compromising overall environmental performance. The sensitivity analysis of electricity consumption and gaseous emissions from composting further highlights the dominant contributors to environmental impacts. The results indicate that CCOF is particularly sensitive to composting emissions: carbon footprint, acidification, and terrestrial eutrophication vary by 12∼19% under a ± 20% variation in composting emissions. In contrast HMDOF is more strongly influenced by electricity use, which all the considered environmental impacts varying by 8∼17% under a ±20% change in electricity use. For HMDOF, fossil resource depletion is identified as the most affected category driven the higher electricity demand of automated machinery. Adopting more automated or intelligent equipment can improve processing efficiency; however, the additional electricity required to operate these systems may result in higher emissions, especially when the electricity mix is still dominated by fossil fuels. These findings highlight the importance of integrating circular agriculture from a new perspective, as well as the fundamental trade-off between process efficiency and environmental performance in composting technologies.

## CRediT authorship contribution statement

**Yu-Ning Chen:** Writing – review & editing, Writing – original draft, Methodology, Investigation. **Yen-Ching Lin:** Investigation, Data curation. **Shu-Yuan Pan:** Writing – review & editing, Writing – original draft, Funding acquisition, Data curation, Conceptualization. **Ching-Hsun Chang:** Writing – review & editing, Conceptualization. **Tsai-Chi Kuo:** Validation, Methodology, Investigation, Conceptualization.

## Disclosures

The authors declare that they have no known competing financial interests or personal relationships that could have appeared to influence the work reported in this paper.
